# A Machine‐Learning Model of Chronological Age Based on Routine Blood Biomarkers in a Central European Population: A Potential Biological Age Marker

**DOI:** 10.1155/jare/9924922

**Published:** 2025-12-31

**Authors:** Pavel Borsky, Drahomira Holmannova, Tereza Maresova, Anabela Cizkova, Tereza Kempfova, Svatopluk Byma, Tom Philipp, Lenka Borska

**Affiliations:** ^1^ Department of Preventive Medicine, Faculty of Medicine in Hradec Kralove, Charles University, Hradec Kralove, Czech Republic, cuni.cz; ^2^ Synlab Czech s.r.o., Prague, Czech Republic; ^3^ Nephrology Clinic, University Hospital Hradec Kralove and Faculty of Medicine in Hradec Kralove, Charles University, Hradec Kralove, Czech Republic, cuni.cz; ^4^ Clinic of Rheumatology and Physiotherapy, Third Faculty of Medicine, Charles University and Thomayer University Hospital, Prague, Czech Republic, cuni.cz

**Keywords:** age, aging, AI, biomarkers, blood

## Abstract

**Background:**

Aging is a gradual decline in physiological and functional capacities that leads to an exponentially increasing risk of death. Although aging is universal, the rate of aging differs substantially between individuals. Biomarkers of aging are being developed to improve the prediction of a person’s susceptibility to disease onset, disease course, and complications, as well as to estimate lifespan and healthspan.

**Objective:**

The primary aim of this study was to develop and evaluate machine‐learning models that estimate chronological age from routinely measured blood biomarkers in a large Central European population. A secondary aim was to characterize the relative contribution of individual biomarkers and to discuss the resulting index as a potential biological age marker.

**Methods:**

We modeled chronological age as a regression problem using four algorithms: a multilayer neural network, Extreme Gradient Boosting (XGBoost), Random Forest, and Ridge Regression. The dataset comprised more than 26 million anonymized laboratory results from over 3 million individuals. Model performance was assessed using mean absolute error (MAE), root mean squared error (RMSE), mean absolute percentage error (MAPE), and epsilon‐accuracy. We also examined feature importance to identify the most informative biomarkers.

**Results:**

XGBoost achieved the best performance, with an MAE of 8.73 years across all ages. The 10 most influential predictors were alanine aminotransferase (ALT), creatinine, alkaline phosphatase (ALP), glucose, mean corpuscular volume (MCV), thrombocytes, albumin, mean corpuscular hemoglobin (MCH), urea, and aspartate aminotransferase (AST). These markers span hepatic, renal, metabolic, and hematological domains.

**Conclusion:**

Using easily accessible blood biomarkers, it is possible to estimate chronological age with an MAE of 8.73 years in a large Central European population. Because the present work does not include validation against clinical outcomes, the resulting index should be regarded as a potential biological age marker. Future studies are needed to test its association with morbidity, mortality, and established biological age measures in independent cohorts.

## 1. Introduction

Aging is the steady deterioration in the physiological and functional capacities of the human body, impacting all organ systems, tissues, and cellular processes. While chronological age measures the passage of time since birth, it does not account for the biological variability of aging, as people of the same chronological age might have considerably different health trajectories and morbidity and mortality risks [1, 2].

The “Hallmarks of Aging” provide a framework for understanding the molecular and cellular mechanisms that underpin aging. These hallmarks are categorized as interrelated processes, including genomic instability, mitochondrial failure, and cellular senescence. The processes in question vary considerably among individuals and are influenced by genetic predisposition, lifestyle choices, and environmental exposures [3].

In light of these discrepancies, there is a growing attempt to identify biomarkers that better reflect biological age. One of the most promising ways is the use of blood‐based biomarkers, which are frequently tested in clinical practice and provide a practical, cost‐effective alternative to more advanced but less accessible procedures such as epigenetic clocks and telomere length evaluations.

The most well known of these, Horvath’s epigenetic clock, may predict biological age with amazing precision but needs costly DNA methylation analysis. Functional tests, while clinically important, frequently fail to capture systemic aging completely. Thus, readily available biochemical markers provide a tempting middle ground [4, 5].

Existing biological age models, including PhenoAge and the Klemera–Doubal method (KDM), have shown predictive power in selected populations. However, these models are often limited by assumptions of linearity, predefined biomarker panels, or smaller sample sizes. Our work builds on these foundations by using a large‐scale, real‐world clinical dataset and contemporary machine‐learning techniques to model age‐related variation in routine blood biomarkers [6].

In the present study, our primary goal was to use commonly acquired blood biomarkers to construct and compare machine‐learning models for the prediction of chronological age. Our secondary goal was to assess the relative contribution of individual biomarkers and to explore whether the resulting index could be considered a potential blood‐based marker of biological age, acknowledging that formal validation against clinical outcomes is not yet available.

In contrast to predefined mathematical formulas or resource‐intensive approaches, such as epigenetic clocks, this study relies on data‐driven machine‐learning methods. These methods can capture complex, nonlinear relationships between routinely assessed hematological, metabolic, and liver and kidney function parameters.

The overarching aim is to provide a scalable modeling framework and a minimal panel of routinely measured biomarkers that, after appropriate longitudinal validation, could be integrated into clinical workflows as an accessible tool for age‐related risk stratification and monitoring of geroprotective interventions.

## 2. Materials and Methods and Results

### 2.1. Data and Preprocessing

**Figure 1:**
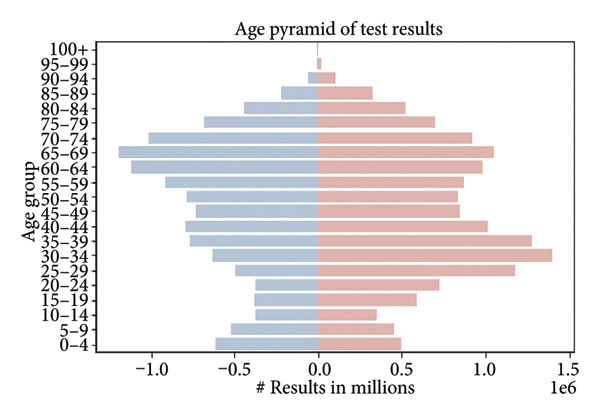
Target variable—participants by age. Displayed as an age pyramid. The population pyramid illustrates the distribution of test results by age group and gender. Each bar represents the number of test results (in millions) within a specific age category, with males shown on the left (in blue) and females on the right (in red). The highest concentration of test results is observed in the middle‐aged groups (35–69 years), with a noticeable decline in both younger (under 20) and older (over 85) age categories. Target variable: Age of an individual, measured as the number of years from birth to the time of sampling.

The dataset under consideration consists of anonymized clinical test results collected from a Central European population, primarily between 2010 and 2020. The database contains over 26 million individual records from more than 3 million individuals. Each record corresponds to a single blood test result, and multiple samples per person are common (median = 4 samples; 90% of individuals have fewer than 19 samples) (Figure 1).

**Figure 3:**
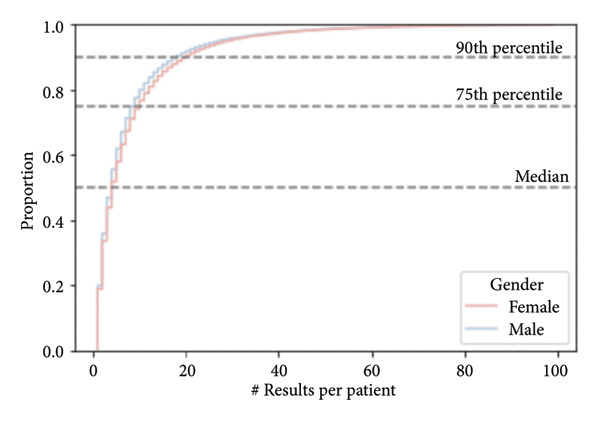
Number of samples per patient. Cumulative distribution functions (CDFs) of the number of results per patient stratified by gender (female—red line, male—blue line). The dashed horizontal lines indicate the median (50th percentile), 75th percentile, and 90th percentile thresholds. The curves illustrate the proportion of individuals with values less than or equal to a given value, allowing visual comparison of distributional differences between genders.

The population is approximately balanced in terms of sex, with 1,688,886 females and 1,511,453 males. Certain age cohorts exhibit imbalances, with a higher number of younger females, likely due to increased access to reproductive health screening, and fewer males in older age groups, reflecting demographic trends (Figures 2 and 3).

Figure 2Distribution by year of birth. (a) Kernel density estimate, (b) normalized by year. There are three major peaks in the density (a), representing temporal booms in the observed population. Normalization by year (b) shows variation in gender distribution by year.

**Figure:**
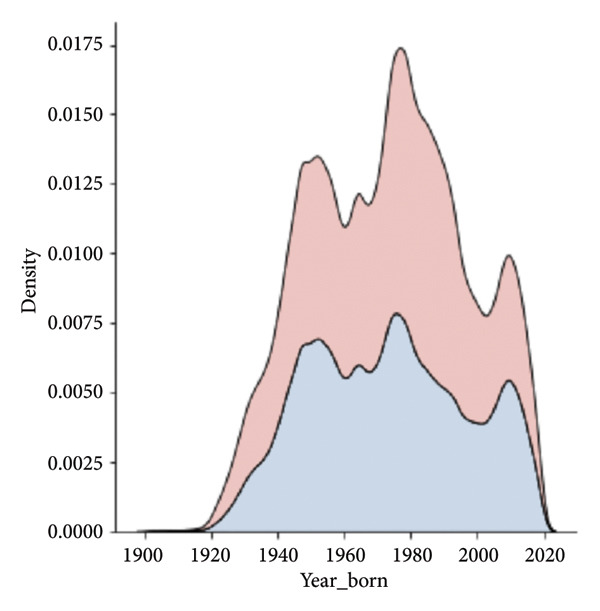
(a)

**Figure:**
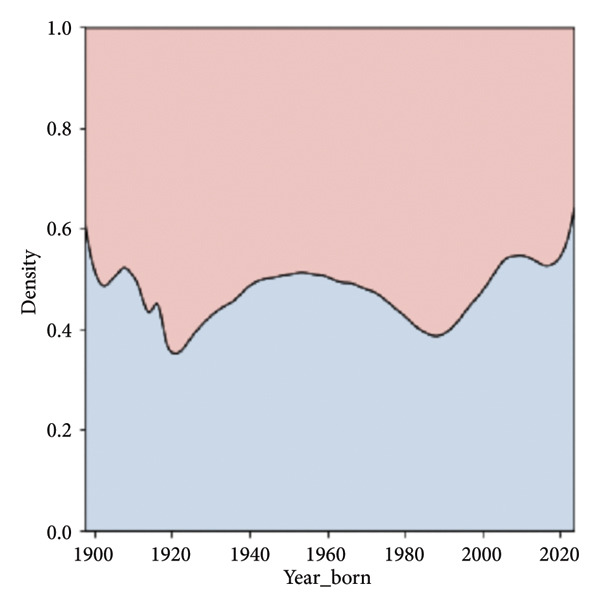
(b)

**Figure 4:**
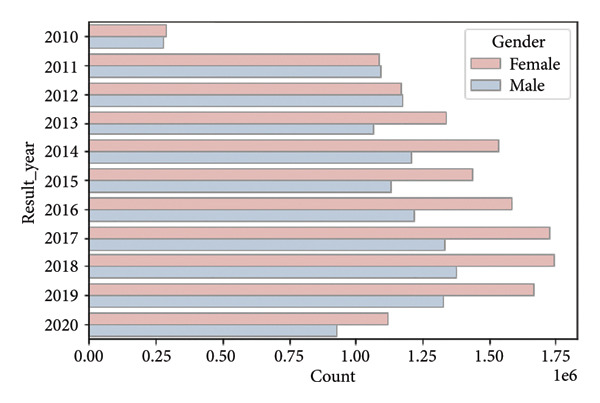
Number of results per year, grouped by gender.

There are 26,818,974 test results in the dataset for valid individuals, predominantly between 2010 and 2020 (Figure 4).

Because the dataset is fully anonymized and provided at the level of individual laboratory reports, we retained all available samples and treated each record as an independent observation of an age–biomarker profile. This design maximizes the amount of information on age‐related biomarker trajectories, but it also introduces a potential sampling bias: individuals with chronic diseases may contribute more samples than healthy individuals who are tested only occasionally. We explicitly acknowledge this limitation, and its implications in the Discussion section.

The dataset includes results from individuals across the full lifespan. However, due to nonlinear developmental changes in childhood and adolescence, the marker is not intended for use below 20 years of age, and predictions in this age range should be interpreted with caution. In the Discussion section, we consider the possibility of developing a separate marker for younger age groups as a future research direction.

To address the issue of missing data, a filtering strategy was implemented. An initial feature‐importance analysis using XGBoost with Gini impurity scores identified the 10 most predictive biomarkers. We retained only records with complete data for all 10 parameters, thereby avoiding the need for imputation and the potential bias associated with different imputation strategies. While this reduced the size of the analytical dataset, it ensured consistency and robustness across models.

### 2.2. Machine Learning Models

We trained and evaluated four machine learning models:

1. XGBoost (Extreme Gradient Boosting): A tree‐based ensemble method known for handling nonlinear relationships, robustness to outliers, and high predictive accuracy.
2. Random Forest (RF): An ensemble of decision trees trained on bootstrapped samples to mitigate overfitting.
3. Ridge Regression: A linear model with L2 regularization, providing a baseline comparison.
4. Neural Network (NN): A multilayer perceptron trained with backpropagation.

Model training was conducted using 5‐fold cross‐validation on an 80:20 train–test split. Performance was evaluated using the following metrics:

• Mean absolute error (MAE)
• Root mean squared error (RMSE)
• Mean absolute percentage error (MAPE)
• Epsilon‐accuracy (the proportion of predictions within a specified error margin)

While Ridge regression provided a simple linear benchmark, XGBoost outperformed all other models across metrics (MAE = 8.73 years), demonstrating its suitability for capturing complex interactions in aging‐related biomarkers.

Future extensions include the exploration of kernel‐based models (e.g., Support Vector Regression with RBF kernels) and ensemble strategies that stack predictions from multiple models, including deep learning architectures.

The final set of 10 biomarkers employed in the modeling process includes alanine aminotransferase (ALT), creatinine, alkaline phosphatase (ALP), glucose, mean corpuscular volume (MCV), thrombocytes, albumin, mean corpuscular hemoglobin (MCH), urea, and aspartate aminotransferase (AST). These markers span the hepatic, renal, metabolic, and hematological domains, offering a broad physiological representation (Table 1).

**Table 1:** Feature importance of the most influential parameters, XGBoost model, measured by Gini impurity.

	Gini impurity (importance)	Samples
ALT	**2817**	4,456,008
Creatinine	**2628**	4,281,418
ALP	**2429**	2,195,932
Glucose	**2297**	4,652,202
MCV	**1743**	5,467,194
Thrombocytes	**1738**	5,410,333
Albumin	**1734**	773,885
MCH	**1734**	5,487,006
Urea	**1661**	3,726,174
AST	**1601**	4,320,589

*Note:* Gini impurity (importance) values denote the feature importance derived from the random forest, computed as the (mean) decrease in Gini impurity attributable to splits on a given variable (also called mean decrease in impurity, MDI), aggregated across all trees. Larger values indicate a greater contribution of the variable to reducing node impurity (i.e., higher predictive/discriminative influence in the model).

Visualizations of biomarker trends across age (e.g., creatinine, urea, and C‐reactive protein [CRP]) are provided to illustrate the biological plausibility of their age association. These findings serve to substantiate the rationale underlying their inclusion prior to the modeling process.

**Figure 5:**
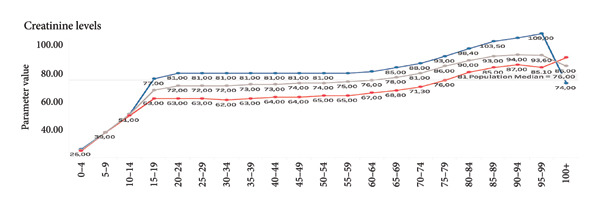
Creatinine. The line chart displays average creatinine levels across different age groups, separated by gender. The blue line represents males, the red line represents females, and the gray line indicates the overall population median. Creatinine values rise sharply in early childhood, stabilizing during adolescence and early adulthood. From around the age of 50, levels gradually increase. Levels in females rise throughout life, while males over 90 years of age are experiencing a rapid decline in creatinine levels.

**Figure 6:**
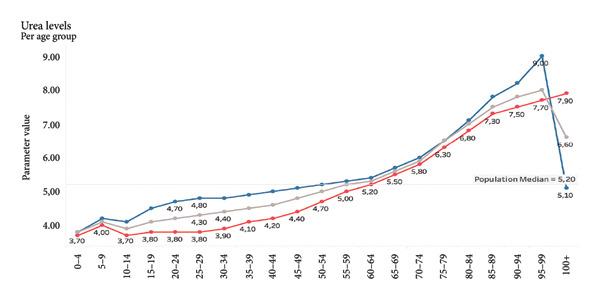
Urea. The line graph shows the average urea levels across different age groups, with a breakdown by gender. Male values are shown in blue, female values in red, and the gray line represents the overall population median. Urea levels remain relatively stable through childhood and early adulthood, and then gradually increase with age. The rise is more pronounced after the age of 60, particularly in males. In individuals over 90, there is a noticeable peak in males, after that, the levels decrease in males, while in females, there is still a slight increase in values.

**Figure 7:**
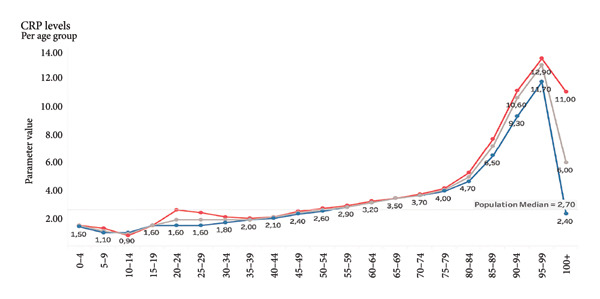
CRP. The line chart presents average CRP levels across age groups, separated by gender. The red line represents females, the blue line represents males, and the gray line marks the overall population median. CRP levels are relatively stable throughout most of life, with only minor fluctuations. However, a sharp increase is observed in older age groups, particularly after age 80. The highest values are seen in individuals aged 95–99. Notably, CRP levels are generally slightly higher in females than in males across most age groups. In the oldest groups, levels of CRP sharply decrease.

**Figure 8:**
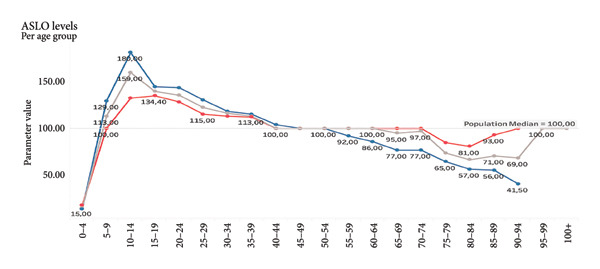
ASLO. The line chart illustrates average antistreptolysin O (ASLO) levels across age groups, with values shown separately for males (blue) and females (red). The gray line represents the population median. ASLO levels rise sharply during childhood, peaking in the 10–14 age group (with a maximum around 180 IU/mL). From young adulthood onward, ASLO levels gradually decline with age. In older adults (75+), levels drop, reaching their lowest in the 95–99 age group but only in males. Levels slightly rise in females.

**Figure 9:**
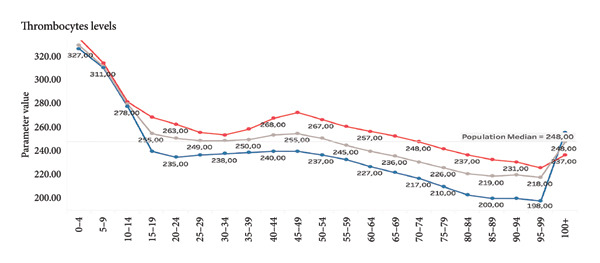
Thrombocytes. The line graph displays average platelet counts across different age groups, differentiated by gender. Female values are shown in red, male values in blue, and the population median is indicated by the gray line. Platelet levels are highest in early childhood, peaking in the 0–4 age group (above 320 × 10^9^/L), and steadily decrease with age. Values in females remain consistently higher than male values across all age groups. In the elderly (especially after age 75), platelet counts decrease more markedly in males. In the oldest, levels of thrombocyte rapidly increase, mainly in males.

For demonstration purposes, we show selected examples of parameters that correlate positively with age. Selected parameters *positively* correlate with age (Figures 5, 6, and 7). Selected parameters *negatively* correlate with age (Figures 8 and 9).

### 2.3. Software and Implementation

All analyses were performed using Python (Version 3.9) with the scikit‐learn and XGBoost libraries. Data preprocessing and visualization were carried out in Python and R (Version 4.1). The full implementation of the biological age algorithm (Python script and trained model) will be released in a public code repository within 12 months after publication of this article. In the interim, the implementation will be made available by the corresponding author upon reasonable request for noncommercial academic use.

## 3. Results

Table 2 summarizes the performance of all four models. XGBoost achieved the lowest MAE and RMSE, along with the highest epsilon‐accuracy, indicating superior predictive power across the tested algorithms.

**Table 2:** Among these models, XGBoost achieved the best performance, with the lowest MAE and the highest accuracy across all evaluation metrics.

Model	MAE	RMSE	MAPE	Epsilon‐accuracy
NN	9.80	61,847	0.59	0.28
XGBoost	**8.73**	50,622	0.54	0.38
RF	8.89	52,681	0.55	0.37
Ridge	9.63	59,869	0.57	0.29

*Note:* MAE and RMSE summarize the average prediction error (lower is better), MAPE expresses the average error as a percentage of the true value (lower is better), and ε‐accuracy is the proportion of predictions that fall within a predefined tolerance (higher is better). XGBoost, XGBoost model; Ridge, Ridge regression.

Abbreviations: NN, neural network; RF, random forest.

In addition, feature importance analysis confirmed that creatinine, ALT, ALP, glucose, and albumin were the most influential predictors. Their biological relevance is supported by prior literature linking these parameters to age‐related physiological changes.

## 4. Discussion

The present study developed and evaluated machine‐learning models for estimating chronological age from commonly available blood biomarkers. Among the four algorithms tested, the XGBoost model achieved the best predictive performance, with an MAE of 8.73 years. The 10 most influential biomarkers—ALT, creatinine, ALP, glucose, MCV, thrombocytes, albumin, MCH, urea, and AST—span a range of physiological domains, including hepatic, renal, metabolic, and hematologic systems.

For example, rising creatinine and urea levels with age reflect declining renal function, while decreasing platelet counts and shifts in MCV/MCH suggest hematologic aging processes. This biological plausibility reinforces the validity of our model. However, because our models were trained on chronological age rather than directly on age‐related clinical outcomes, the resulting index should be interpreted as a chronological age–based biomarker with potential relevance to biological age, rather than as a fully validated biological age measure. Formal validation against age‐related phenotypes (e.g., incident disease, frailty, and mortality) and head‐to‐head comparison with established indices such as PhenoAge and KDM in the same dataset remain important next steps.

The XGBoost algorithm has already been used to train data in the past using different parameters. This algorithm was used, e.g., for prediction of neurological recovery after spinal cord injury, diabetes onset, survival of patients with gliomas, mortality after trauma, mortality in ICU, risk of stroke, mortality in the elderly, aging of blood vessels, and others [7–10].

This model was also used by Bae et al., who used the health check‐up data of more than 111,000 subjects and 35 variables. They compared traditional statistical methods and four AI techniques (RF, XGB, SVR, and DNN) for the prediction of biological age. The results revealed that AI models outperformed traditional statistical methods in predicting biological age [11].

Our model performs comparably to established biological age metrics such as PhenoAge (combines multiple clinical metrics such as chronological age, albumin, creatinine, CRP, ALP, glucose, lymphocyte percentage, MCV, red blood cell distribution width (RDW), and white blood cell count) and the KDM, which have demonstrated MAEs in the range of 5–7 years in more selected populations. Systemic review by Zurbuchen et al. showed that PhenoAge and KDM are strongly validated and precisely predict biological age, mortality, and KDM as well as healthspan. The multiple linear regression, principal component analysis, and Hochschild’s method were not so precise [6, 12, 13].

It is important to acknowledge that these models frequently utilize smaller datasets or operate under the assumption of linearity between predictors and age. In contrast, the findings of our study are supported by a large dataset, comprising over 26 million test results from more than 3 million individuals. The minor increase in MAE in our model may be suggestive of the intricacy and heterogeneity that is inherent in authentic clinical data. The results of the present study indicated that the implementation of machine learning models for the purpose of estimating biological age from blood‐based biomarkers constitutes an efficient method. Among the various models evaluated, XGBoost demonstrated the most optimal overall performance. In comparison to established models such as PhenoAge and KDM, which report MAE values ranging from 5 to 7 years, our model presents a competitive alternative, particularly in consideration of its scalability and practical implementation in clinical laboratories. Despite the slight increase in MAE, the model’s enhanced interpretability and explainability through feature importance emerges as a key advantage, particularly when considering the broader and more heterogeneous characteristics of the population. It is imperative to acknowledge that the dataset under consideration is more than 100 times larger than those typically utilized in benchmark studies. This substantial augmentation in size serves to enhance the generalizability of the findings.

One of the most comparable models is Aging.AI, a neural network‐based predictor that also use the biomarkers obtained from blood. Aging.AI has reported an MAE in the range of 5–7 years [14]. Our model demonstrates a slightly higher MAE, but benefits from explainability through feature importance analysis. In our study, creatinine, ALT, ALP, glucose, and albumin emerged as key contributors to biological age estimation, in agreement with previous research highlighting the importance of metabolic, renal, and hepatic biomarkers in aging processes. It should also be noted that the number of individual results being used in our model is approximately hundred times higher; therefore, it may be argued that lower MAE in the study of Aging.AI might be statistically more biased than ours [15].

When comparing the use of XGBoost and other models, this algorithm has advantages in prediction of biological age. The advantage of the complex XGBoost‐based model was the higher predictive accuracy of the risk of death compared with the linear Cox model PhenoAge. Interestingly, a simplified version of ENABL Age‐L using only routine biochemistry (similar markers to PhenoAge but omitting CRP) achieved almost the same predictive power as the full model—suggesting that PhenoAge (which includes CRP) can be replaced by purely routine tests without loss of accuracy. This study directly demonstrates that modern XGBoost/GBTs models can outperform the established phenotypic age of PhenoAge in clinical applications (mortality prediction) while offering interpretability at the individual level (using XAI) as a practical advantage.

Qui et al. showed that XGBoost within the ENABL Age framework enabled accurate, robust, and explainable prediction of biological age better than PhenoAge. With interpretable outputs, the results can be used for personalized medicine and targeted interventions, increasing clinical applicability and physician confidence in AI models [16].

Zhang et al. evaluated different models for biological age prediction in a long living population, including centenarians. The models included both classical approaches—multiple regression, PCA, two variants of Klemer–Doubal (KDM1, KDM2)—and ML algorithms: Random Forest, SVM, XGBoost, and LightGBM. They selected 80 indicators covering nutrition, cardiovascular, liver, kidney, bone, endocrine, hematological, and immune functions, and the performance of the models was evaluated using *R* ^2^, with the best model scoring 0.92 and the worst scoring 0.45. The best performing model in cross‐validation was KDM2 (*R* ^2^ = 0.89), followed by PCA (*R* ^2^ = 0.62). XGBoost was one of the eight models successfully applied but was not the best in predicting the biological age of centenarians compared to the other models. Thus, it appears that in the case of very old age, XGBoost is less accurate in determining biological age than the other groups [17]. Bernard et al. showed XGBoost as the most appropriate algorithm and created an innovative explainable ML framework to determine a personalized physiological age (PPA) with 26 variables; glycated hemoglobin (HbA1c) displays a major relative weight in the estimation of PPA [18].

In summary, XGBoost and other ML algorithms often offer higher accuracy in predicting biological age and related clinical outcomes (mortality, morbidity) than traditional indices such as PhenoAge or KDM. At the same time, however, they emphasize the trade‐off between complexity and interpretability: while PhenoAge/KDM excel in simplicity and proven predictive value, XGBoost‐based models can make better use of nonlinear biomarker information and thus improve performance (lower MAE/RMSE, higher AUC or c‐index) Thus, for the purposes of the user article, it is useful to cite the above studies, which not only use similar biomarkers (ALT, AST, ALP, albumin, glucose, etc.) but directly document the advantages and disadvantages of XGBoost over PhenoAge/KDM in the context of biological age estimation and clinical risk prediction.

Duran et al. revealed that the XGBoost algorithm has also proven successful in the use of transcriptome detection analysis from human blood in the context of age determination. They proved that there is a profile difference in the human polymorphonuclear blood mononuclear cells across a wide age range. The transcriptome was analyzed by LASSO (Least Absolute Shrinkage and Selection Operator) (LASSO), EN (Elastic Net), XGBoost (eXtreme Gradient Boosting), and LightGBM (Light Gradient‐Boosting Machine) algorithms. The most accurate single model was XGBoost—it had the lowest error (MAE ≈ 5.3 years) and the highest correlation with chronological age and LightGBM achieved similar results to XGBoost but was slightly less consistent across datasets. The Ensemble model (average of multiple methods) was the most stable across datasets and combined the strengths of each algorithm. XGBoost allows not only accurate age prediction, but also interpretation of how individual biomarkers contribute to the resulting prediction. This allows the identification of biomarkers that are lifestyle influenced and can be the target of personalized interventions to improve health and longevity [19].

Qomariyah et al. used various algorithms including XBoost and markers from blood (hematology, parameters of coagulation, renal and liver functions, oxygen saturation, cardiac enzymes, etc.) to predict biological age in patients with COVID‐19. The results showed that the XGBoost algorithm can be used to predict the patients’ age from their routine blood tests. The performance evaluation is very satisfactory, with *R* ^2^ > 0.80 and a normalized RMSE below 0.1 [20].

Biological age can be determined using other methods, but these are more expensive and often less accessible than methods that can use common blood markers, which almost any laboratory can determine. Epigenetic clocks, such as Horvath’s model, achieve higher accuracy (MAE ∼3 years) but require costly DNA methylation analysis and are not routinely used in clinical practice [21, 22].

XGBoost can also be used when DNA methylation is used to determine biological age as in the epigenetic clock. Zhou et al. employed XGBoost, LightGBM, CatBoost, and deep neural networks as machine learning models for training to predict biological age from DNA methylation data. XGBoost achieved the lowest error (MAE) and the best agreement with biological age (*R* ^2^) on both training and test sets. For example, on the test set, XGBoost had an MAE of 3.6089 and an *R* ^2^ of 0.9441, while DNN had an MAE of 5.4531 and an *R* ^2^ of 0.9124. CatBoost had a very good match (*R* ^2^ of 0.9974) on the training set, but on the test set, XGBoost is generally the best [23].

Telomere length is another popular aging biomarker, but it suffers from poor reproducibility and high interindividual variability. Functional markers (e.g., grip strength) can be clinically useful but lack the granularity needed for precise age estimation [24–26].

In contrast, our model based on routine blood chemistry provides a cost‐effective and scalable solution, accessible in primary care or preventive screening settings, and capable of real‐time monitoring. The potential application of biological age prediction in personalized medicine is significant, as it could improve early interventions for individuals whose biological age is higher than their chronological age. Such integration could offer particular value in geriatric assessments and preventive care, where the ability to detect accelerated aging early on is very important.

Given the sensitivity of blood biomarkers to lifestyle and therapeutic interventions, our model has the prospective capacity to function as a dynamic monitoring instrument, thereby facilitating the evaluation of the effectiveness of geroprotective therapies, including caloric restriction, senolytics, and anti‐inflammatory strategies [27, 28]. In contrast to epigenetic clocks, which exhibit a more gradual change, blood biomarkers routinely analyzed in laboratories such as fasting glucose, CRP, and ALT have been shown to respond promptly to interventions (see Figures 6 and 7).

This finding supports the integration of biological age assessments into personalized health strategies. XGBoost has three main advantages. First, it can model complex nonlinear relationships. Second, it is robust to outliers. Third, it has adaptive learning mechanisms that emphasize difficult‐to‐predict cases. Studies have shown that XGBoost can be successfully used to predict things like mortality, stroke, and organ‐specific aging, which further supports its use in biomedical contexts. These findings suggest that the XGBoost algorithm belongs among the best options for estimating biological age across different types of biological data. In addition, its ability to work with biomarkers that can change and be affected by treatment, like those found in blood, makes it a good tool for evaluating the aging process in patients and for checking the effects of geroprotective strategies over time.

## 5. Limitations and Future Directions

This work has several important limitations. First, the dataset consisted of anonymized laboratory records without longitudinal clinical follow‐up. As a result, we were not able to validate the index against age‐related outcomes such as incident disease, disability, or mortality, nor could we test whether it adds prognostic value beyond established biological age measures. Consequently, we use the more cautious term “potential biological age marker” throughout the manuscript.

Second, because the data were provided at the level of individual laboratory reports and many individuals had repeated measurements, we treated each record as an independent observation. This choice maximized the available sample size and allowed us to model detailed age‐related trends. However, it also introduces a potential sampling bias: individuals with chronic conditions typically undergo more frequent testing and therefore contribute disproportionately to the dataset compared with healthy individuals. A future reanalysis that restricts modeling to a single (e.g., earliest) measurement per person and uses subsequent measurements for incident‐disease analyses would help to quantify this bias more precisely.

Third, although the dataset covers the entire lifespan, the developmental changes in childhood and adolescence are highly nonlinear. We therefore do not recommend applying the current marker to individuals younger than 20 years of age. Separate models tailored to pediatric and adolescent populations may be warranted.

Fourth, we did not implement a head‐to‐head comparison with established biological age indices such as PhenoAge or KDM in this dataset. Such analyses would require consistent availability of all component variables and substantial additional modeling work. Instead, we compared our performance metrics to those reported in the literature and discussed similarities and differences qualitatively.

Finally, although we used a large, real‐world dataset and contemporary machine‐learning methods, additional external validation in independent cohorts, ideally with detailed clinical outcomes, is needed before the index can be recommended for routine clinical decision‐making. Future work should also explore alternative modeling strategies (e.g., ensemble models, deep learning architectures) and the use of this index as a dynamic marker to monitor the effects of geroprotective interventions.

## 6. Conclusion

We present a robust and interpretable machine‐learning model for estimating chronological age from ten routinely measured blood biomarkers. Our XGBoost‐based approach achieves strong predictive performance (MAE = 8.73 years) and offers clear advantages in terms of clinical accessibility and cost‐effectiveness compared with more complex genetic or epigenetic aging models.

Because the model has not yet been validated against clinical outcomes, it should currently be regarded as a potential biological age marker and a methodological framework rather than as a fully established biological age index. Nevertheless, its reliance on widely available laboratory tests and its ability to capture nonlinear relationships among biomarkers suggest that it could become a useful tool for large‐scale screening, risk stratification, and monitoring of age‐related changes, once further validation is available.

Future research should focus on (i) longitudinal validation of the index with respect to morbidity, disability, and mortality; (ii) head‐to‐head comparisons with existing biological age measures in the same cohorts; and (iii) exploration of its responsiveness to lifestyle and geroprotective interventions. Such studies will clarify whether this routine‐biochemistry‐based index can be integrated into personalized aging medicine as a practical complement to more complex molecular biomarkers.

## Ethics Statement

The study was conducted in accordance with the Declaration of Helsinki, and the protocol was approved by the Ethics Committee of the Faculty Hospital in Hradec Kralove, Czech Republic (Project identification code PROGRES Q40‐09 and Q40‐10, reference number 201705 I83P, date 2 May 2017).

## Consent

The authors have nothing to report.

## Conflicts of Interest

The authors declare no conflicts of interest.

## Funding

The study was supported by Cooperatio UK—Health Sciences and Charles University, Faculty of Medicine in Hradec Kralove, the Czech Republic, project SVV‐2025‐260776.

## Data Availability

The data that support the findings of this study are available upon request from the corresponding author. The data are not publicly available due to privacy or ethical restrictions.

## References

[1] Khan S. S. , Singer B. D. , and Vaughan D. E. , Molecular and Physiological Manifestations and Measurement of Aging in Humans, Aging Cell. (2017) 16, no. 4, 624–633, 10.1111/ACEL.12601;REQUESTEDJOURNAL:JOURNAL:14749726;CTYPE:STRING:JOURNAL. 28544158 PMC5506433

[2] Wang H. , Liu Z. , Fan H. et al., Association Between Biological Aging and the Risk of Mortality in Individuals With Non-Alcoholic Fatty Liver Disease: A Prospective Cohort Study, Archives of Gerontology and Geriatrics. (2024) 124, 10.1016/J.ARCHGER.2024.105477. 38735225

[3] López-Otín C. , Blasco M. A. , Partridge L. , Serrano M. , and Kroemer G. , Hallmarks of Aging: An Expanding Universe, Cell. (2023) 186, no. 2, 243–278, 10.1016/J.CELL.2022.11.001. 36599349

[4] Kuiper L. M. , Polinder-Bos H. A. , Bizzarri D. et al., Epigenetic and Metabolomic Biomarkers for Biological Age: A Comparative Analysis of Mortality and Frailty Risk, The Journals of Gerontology: Series A. (2023) 78, no. 10, 1753–1762, 10.1093/GERONA/GLAD137. PMC10562890 37303208

[5] Pearce E. E. , Alsaggaf R. , Katta S. et al., Telomere Length and Epigenetic Clocks as Markers of Cellular Aging: A Comparative Study, GeroScience. (2022) 44, no. 3, 1861–1869, 10.1007/S11357-022-00586-4/TABLES/3. 35585300 PMC9213578

[6] Zurbuchen R. , von Däniken A. , Janka H. , von Wolff M. , and Stute P. , Methods for the Assessment of Biological Age – a Systematic Review, Maturitas. (2025) 195, 10.1016/J.MATURITAS.2025.108215. 39938306

[7] Shin H. , Noh G. , and Choi B. M. , Photoplethysmogram Based Vascular Aging Assessment Using the Deep Convolutional Neural Network, Scientific Reports. (2022) 12, 1–10, 10.1038/S41598-022-15240-4;SUBJMETA=139. 35790836 PMC9256729

[8] Noh B. , Youm C. , Goh E. et al., Xgboost Based Machine Learning Approach to Predict the Risk of Fall in Older Adults Using Gait Outcomes, Scientific Reports. (2021) 11, 1–9, 10.1038/S41598-021-91797-W. 34108595 PMC8190134

[9] Khurshid M. R. , Manzoor S. , Sadiq T. , Hussain L. , Khan M. S. , and Dutta A. K. , Unveiling Diabetes Onset: Optimized Xgboost With Bayesian Optimization for Enhanced Prediction, Plos One. (2025) 20, no. 1, 10.1371/JOURNAL.PONE.0310218. PMC11760023 39854291

[10] Tan M. L. , Ruffle D. J. , Mohinta M. S. et al., Mathematical Modelling of Survival in Low Grade Glioas at Malignant Transformation With Xgboost, Neuro-Oncology. (2024) 26, vii12–vii13, 10.1093/NEUONC/NOAE158.048.

[11] Bae C.-Y. , Im Y. , Lee J. et al., Comparison of Biological Age Prediction Models Using Clinical Biomarkers Commonly Measured in Clinical Practice Settings: AI Techniques Vs. Traditional Statistical Methods, Frontiers in Analytical Science. (2021) 1, 10.3389/FRANS.2021.709589.

[12] Klemera P. and Doubal S. , A New Approach to the Concept and Computation of Biological Age, Mechanism of Ageing and Development. (2006) 127, no. 3, 240–248, 10.1016/J.MAD.2005.10.004, 2-s2.0-32244445440. 16318865

[13] Levine M. E. , Lu A. T. , Quach A. et al., An Epigenetic Biomarker of Aging for Lifespan and Healthspan, Aging. (2018) 10, no. 4, 573–591, 10.18632/AGING.101414, 2-s2.0-85046851006. 29676998 PMC5940111

[14] Mamoshina P. , Kochetov K. , Putin E. et al., Population Specific Biomarkers of Human Aging: A Big Data Study Using South Korean, Canadian, and Eastern European Patient Populations, Journals of Gerontology: Series A Biological Sciences and Medical Sciences. (2018) 73, no. 11, 1482–1490, 10.1093/GERONA/GLY005, 2-s2.0-85050306913. 29340580 PMC6175034

[15] Consortium A. B. , Jiang M. , Zheng Z. et al., A Biomarker Framework for Liver Aging: The Aging Biomarker Consortium Consensus Statement, Life Medicine. (2024) 3, no. 1, 10.1093/LIFEMEDI/LNAE004. PMC11749002 39872390

[16] Qiu W. , Chen H. , Kaeberlein M. , and Lee S. I. , Explainable Biological Age (ENABL Age): An Artificial Intelligence Framework for Interpretable Biological Age, Lancet Healthy Longev. (2023) 4, no. 12, e711–e723, 10.1016/S2666-7568(23)00189-7. 37944549

[17] Zhang W. , Li Z. , Niu Y. et al., The Biological Age Model for Evaluating the Degree of Aging in Centenarians, Archives of Gerontology and Geriatrics. (2024) 117, 10.1016/J.ARCHGER.2023.105175. 37688921

[18] Bernard D. , Doumard E. , Ader I. et al., Explainable Machine Learning Framework to Predict Personalized Physiological Aging, Aging Cell. (2023) 22, no. 8, 10.1111/ACEL.13872. PMC10410015 37300327

[19] Duran I. and Tsurumi A. , Evaluating Transcriptional Alterations Associated With Ageing and Developing Age Prediction Models Based on the Human Blood Transcriptome, Biogerontology. (2025) 26, no. 2, 1–16, 10.1007/S10522-025-10216-Z/METRICS. 40186010

[20] Qomariyah N. N. , Purwita A. A. , Astriani M. S. , Dhuny S. , Asri A. , and Kazakov D. , An Xgboost Model for Age Prediction From CoviD-19 Blood Test, Ieee. (2021) 16–17.

[21] Horvath S. , DNA Methylation Age of Human Tissues and Cell Types, Genome Biology. (2013) 14, no. 10, 1–20, 10.1186/GB-2013-14-10-R115/FIGURES/9. PMC4015143 24138928

[22] Duan R. , Fu Q. , Sun Y. , and Li Q. , Epigenetic Clock: A Promising Biomarker and Practical Tool in Aging, Ageing Research Reviews. (2022) 81, 10.1016/j.arr.2022.101743. 36206857

[23] Zhou S. , Chen J. , Wei S. et al., Exploring the Correlation Between DNA Methylation and Biological Age Using an Interpretable Machine Learning Framework, Scientific Reports. (2024) 14, 1–13, 10.1038/S41598-024-75586-9. 39406876 PMC11480495

[24] Ye Q. , Apsley A. T. , Etzel L. et al., Telomere Length and Chronological Age Across the Human Lifespan: A Systematic Review and Meta-Analysis of 414 Study Samples Including 743,019 Individuals, Ageing Research Reviews. (2023) 90, 10.1016/J.ARR.2023.102031. PMC10529491 37567392

[25] Martens D. S. , Van Der Stukken C. , Derom C. , Thiery E. , Bijnens E. M. , and Nawrot T. S. , Newborn Telomere Length Predicts Later Life Telomere Length: Tracking Telomere Length From Birth to Child- and Adulthood, EbioMedicine. (2021) 63, 10.1016/J.EBIOM.2020.103164/ATTACHMENT/54B18408-8DBE-4518-9A19-B930454A5ECE/MMC1.PDF. PMC7808927 33422989

[26] Vaiserman A. and Krasnienkov D. , Telomere Length as a Marker of Biological Age: State-Of-The-Art, Open Issues, and Future Perspectives, Frontiers in Genetics. (2021) 11, 10.3389/FGENE.2020.630186. PMC7859450 33552142

[27] Landsberger T. , Amit I. , and Alon U. , Geroprotective Interventions Converge on Gene Expression Programs of Reduced Inflammation and Restored Fatty Acid Metabolism, GeroScience. (2023) 46, no. 2, 1627–1639, 10.1007/S11357-023-00915-1. 37698783 PMC10828297

[28] Rivero-Segura N. A. , Zepeda-Arzate E. A. , Castillo-Vazquez S. K. et al., Exploring the Geroprotective Potential of Nutraceuticals, Nutrients. (2024) 16, no. 17, 10.3390/NU16172835. PMC11396943 39275153

